# Association between diastolic blood pressure during the first 24 h and 28-day mortality in patients with septic shock: a retrospective observational study

**DOI:** 10.1186/s40001-023-01315-z

**Published:** 2023-09-09

**Authors:** Zhiwei Gao, Cong Li, Hui Chen, Dongyu Chen, ShaoLei Ma, Jianfeng Xie, Changde Wu, Ling Liu, Yi Yang

**Affiliations:** 1 Jiangsu Provincial Key Laboratory of Critical Care Medicine, Department of Critical Care Medicine, Zhongda Hospital, School of Medicine, Southeast University, Nanjing, 210009 China; 2 Department of Emergency Intensive Care Unit, The Affiliated Huaian NO. 1 People’s Hospital of Nanjing Medical University, Huai’an, 223300 China

## Abstract

**Background:**

Although the mean arterial pressure (MAP) target of 65 mmHg was achieved, diastolic blood pressure (DBP) was still low in some septic shock patients. The effects of DBP on the prognosis and optimal target for patients with septic shock are unclear. We sought to investigate the relationship between DBP and 28-day mortality in septic shock patients.

**Methods:**

In this retrospective observational study, we obtained data from the Chinese Database in Intensive Care (CDIC). We included patients with an admission diagnosis of septic shock and shock was controlled. DBP was measured every 1 h, and the mean DBP during the first 24 h (mDBP_24h_) was recorded. The primary outcome was 28-day mortality. Multivariable logistic regression determined the relationship between mDBP_24h_ and 28-day mortality.

**Results:**

In total, 1251 patients were finally included. The 28-day mortality of included septic shock patients was 28.3%. The mDBP_24h_, not mSBP_24h_, was higher among 28-day survivors compared with non-survivors. 28-day mortality was inversely associated with mDBP_24h_ (unadjusted OR 0.814 per 10 mmHg higher mDBP_24h_, *P* = 0.003), with a stepwise increase in 28-day mortality at lower mDBP_24h_. The 28-day mortality of patients with mDBP_24h_ < 59 mmHg had an absolute risk reduction of 9.4% (*P* = 0.001). And mDBP_24h_ < 59 mmHg was the remaining high risk factor inversely associated with 28-day mortality after multivariable adjustment (adjusted OR 1.915, 95% CI 1.037–3.536, *P* = 0.038), while mMAP_24h_ and mSBP_24h_ were not.

**Conclusion:**

In patients with septic shock after initial resuscitation, we observed an inverse association between mDBP_24h_ and 28-day mortality. The poor outcomes in patients with mDBP_24h_ < 59 mmHg provide indirect evidence supporting a further DBP goal of 59 mmHg for patients with septic shock after MAP of 65 mmHg was achieved.

**Supplementary Information:**

The online version contains supplementary material available at 10.1186/s40001-023-01315-z.

## Background

Septic shock is the most common form of circulatory shock in intensive care units [1]. And septic shock is considered a leading causes of death for critical patients worldwide [2]. A cross-section survey study of forty-four ICUs in mainland China showed that septic shock accounted for 53.3% of all sepsis patients, while 90-day mortality was up to 51.94% [3]. Thus, the surviving sepsis campaign bundle including fluid resuscitation are the most important therapeutic measures to ensure adequate tissue perfusion and prevent poor outcomes in patients with septic shock [4–6]. Initially, maintaining a mean arterial pressure (MAP) greater than 65 mmHg as part of the early fluid resuscitations has been always recommended by the surviving sepsis campaign guidelines [4, 6]. However, even then the target of MAP was achieved, the mortality of septic patients was high [7, 8], indicating that simply reaching the MAP target value is inadequate.

Although a MAP of 65 mmHg was achieved, some patients with septic shock had low diastolic blood pressure (DBP) [9, 10]. DBP is a good marker of vascular tone and upstream pressure for the coronary perfusion. It has been confirmed that low level of DBP, not systolic blood pressure and MAP, was the independent predictor of early progression to septic shock [11], associated with the development of acute kidney injury (AKI) [12, 13], and significantly associated with in-hospital mortality [14, 15]. DBP has been recommended as a trigger to start norepinephrine (NE) treatment while cooperated with MAP in the early resuscitation of septic shock [16, 17]. Considering the clinical relationships between the DBP level and sepsis progression, maintaining a suitable DBP level could be crucial and have immediate effect on prognosis in patients with septic shock.

Given the lack of clinical evidence of specific DBP target levels in septic shock patients, we sought to describe the relationship between DBP and 28-day mortality among patients with septic shock. We hypothesized that 28-day mortality among patients with septic shock would increase as a function of lower DBP and that a threshold DBP may be identified as an optimal DBP range.

## Methods

### Study population

We conducted a retrospective observational study in which the data were extracted from the Chinese Database in Intensive Care (CDIC). The latest CDIC contains about 7,000 admitted to the Department of Crit Care Medicine, Zhongda Hospital, Southeast University, China, from January 2016 to July 2022. Patients in CDIC with septic shock diagnosis within 24 h after ICU admission and shock control were eligible for inclusion. The diagnosis of septic shock was consistent with the third international consensus definitions for sepsis and septic shock (Sepsis-3) [18]. Shock control was defined as achievement of sustained mean arterial blood pressure of at least 65 mmHg, together with urine flow at least 0.5 ml/kg/h for two consecutive hours, or decreased serum lactate great than or equal to 10% from baseline by 6 h after septic shock diagnosis [19]. We only included the first intensive Care Unit (ICU) admission of each patients and excluded patients younger than 18 years, died in the first 24 h after ICU admission, accompanied by moderate or severe aortic valve insufficiency.

The present study was approved by the Research Ethics Commission of Zhongda Hospital Southeast University which certified that the present study was performed in accordance with all required guidelines and regulations (2023ZDSYLL004-P01).

### Data collection and outcome

All demographic data including age, gender, source of infection, chronic comorbidities, vital sign, laboratory, clinical and outcome data were collected. We included the worst values of laboratory test data in the first 24 h after septic shock admission diagnosis. Vital signs containing DBP, systolic blood pressure (SBP), MAP, heart rate (HR), and central venous pressure (CVP) were all recorded every 1 h. Blood pressure of septic shock patients was preferentially recorded from invasive arterial blood pressure monitoring methods, and otherwise from noninvasive methods. The mean DBP during the first 24 h (mDBP_24h_) was calculated as the mean recorded values of the first 24 h after septic shock admission diagnosis. The other vital signs (mMAP_24h_, mSBP_24h_, mHR_24h_, mCVP_24h_) calculation methods are the same as mDBP_24h_.

The vasoactive-Inotropic Score (VIS) was calculated by peak vasopressor and inotrope doses during the first 24 h of septic shock diagnosis (in mcg/kg/min): VIS = dobutamine + dopamine + (10 * phenylephrine + milrinone) + (100 * [epinephrine + norepinephrine]) + (10,000 * units/kg/min vasopressin). And one VIS is considered equal to 1 mcg/kg/min of dobutamine or dopamine or 0.01 mcg/kg/min of epinephrine or norepinephrine [20].

The primary outcome in the CDIC derivation was 28-day mortality. We also recorded other outcome data, such as new mechanical ventilation (MV) and continuous renal replacement therapy (CRRT) during ICU stay, length of ICU and hospital stay, ICU and hospital mortality.

### Statistical analysis

Continuous variables are presented as medians [interquartile ranges (IQRs)] and the Mann–Whitney *U* test was used for comparison in groups. Categorical variables are expressed as number (percentage), and Pearson *χ*^2^ test is used to compare between groups. The number of missing or censoring values are presented in Additional file 1: Table S1. Variables with more than 25% missing ratio were excluded [21]. Outliers were censored, and missing data of less than 25% were replaced with the sequence mean value.

Logistic regression was used to find the association between mDBP_24h_ and 28-day mortality before and after adjusting for age, gender, Acute Physiology and Chronic Health Evaluation II (APACHE II), peak VIS of the first 24 h after ICU admission. We use the area under the receiver-operator characteristic (AUC, *c*-statistic) value, and use the Youden’s* J* index to define the optimal cutoff value. The 28-day survival was evaluated using the Kaplan–Meier survival analysis and Cox proportional-hazards analysis. Two-tailed *P*-value < 0.05 was considered as statistical significance. Analyses were performed by IBM SPSS statistic 25.

## Results

### Study population

**Fig. 1:**
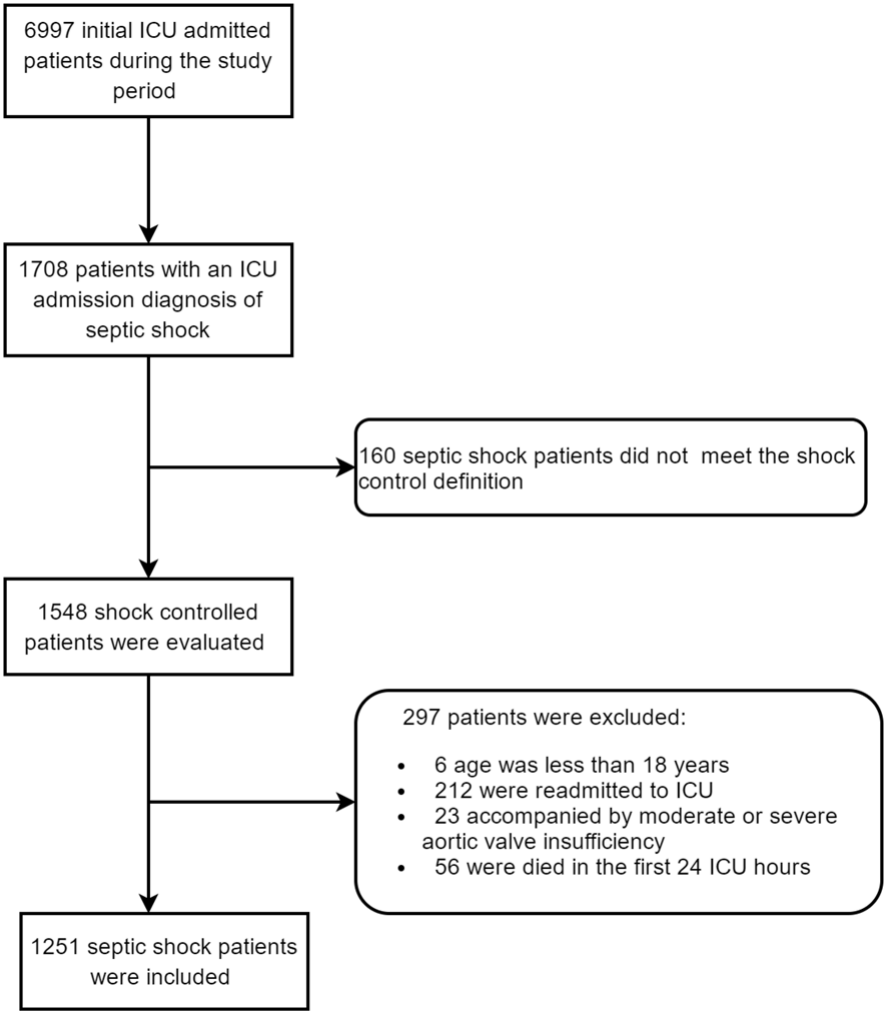
Flow of screening the shock controlled patients after ICU admission of septic shock

The CDIC included 6997 unique ICU patient admissions, and 1548 patients both met septic shock diagnosis and shock control definition, then 297 was excluded due to the exclusion criteria (Fig. 1). The mean age of 1251 patients with septic shock was 68.0 years (55.0–78.0), including 67.5% males. The median Acute Physiology and Chronic Health Evaluation II score (APACHE II) was 19.0 (14.0–25.0). Lung was the leading cause of infection (50.7%).

The 28-day mortality of the septic shock patients enrolled was 28.3%. Compared with septic shock patients in 28-day survival group, the APACHE II and SOFA score were significantly increased in the 28-day non-survival group (*P* < 0.001). The proportion of patients using vasoactive drugs and VIS was similar between the two groups. However mDBP_24h_ and mMAP_24h_ was significantly lower in 28-day non-survival group, while mSBP_24h_ was similar (Table 1).

**Table 1:** Baseline data of septic shock patients between 28 days survival and no-survival groups

	Total *n* = 1251	28-day survival group *n* = 897	28-day non-survival group *n* = 354	*P-*value
Age, median (IQR)	68.0 (55.0–78.0)	67.0 (55.0–77.0)	71.0 (59.3–80.0)	< 0.001
Gender, Male, *n* (%)	845 (67.5)	597 (66.6)	248 (70.2)	0.233
APACHE II score, median (IQR)	19.0 (14.0–25.0)	18.0 (13.0–23.0)	24.0 (19.0–30.0)	< 0.001
SOFA score, median (IQR)	8.0 (5.0–10.0)	7.0 (5.0–10.0)	10.0 (7.0–12.5)	< 0.001
*Comorbidities, n (%)*				
Hypertension	657 (52.5)	466 (52.0)	191 (54.0)	0.523
Diabetes mellitus	336 (26.9)	223 (24.9)	113 (31.9)	0.011
Chronic congestive heart failure	237 (18.9)	158 (17.6)	79 (22.3)	0.059
Coronary heart disease	206 (16.5)	135 (15.1)	71 (20.1)	0.032
Chronic renal failure	93 (7.4)	62 (6.9)	31 (8.8)	0.262
COPD	86 (6.9)	48 (5.4)	38 (10.7)	0.001
Cirrhosis	25 (2.0)	16 (1.8)	9 (2.5)	0.388
Cancer (solid tumor)	237 (18.9)	157 (17.5)	80 (22.4)	0.038
*Source of infection, n (%)*				
Lung	634 (50.7)	415 (46.3)	219 (61.9)	< 0.001
Abdomen	415 (33.2)	320 (35.7)	95 (26.8)	0.002
Blood	47 (3.8)	31 (3.5)	16 (4.5)	0.373
Skin	34 (2.7)	27 (3.0)	7 (2.0)	0.312
Urinary tract	89 (7.1)	80 (9.1)	9 (2.5)	< 0.001
Others	30 (2.4)	22 (2.5)	8 (2.3)	0.841
*Hemodynamic variables, median (IQR)*				
mSBP_24h_ (mmHg)	123.0 (116.6–131.1)	123.2 (116.9–131.0)	122.2 (114.8–129.5)	0.174
mDBP_24h_ (mmHg)	63.3 (57.6–69.3)	63.6 (58.3–69.2)	62.0 (55.7–68.9)	0.003
mMAP_24h_ (mmHg)	83.1 (78.1–88.4)	83.3 (78.6–88.2)	81.6 (76.7–88.0)	0.011
mHR_24h_ (beats per minute)	94.6 (83.0–105.8)	93.0 (82.1–103.3)	100.7 (87.3–112.2)	< 0.001
mCVP_24h_ (mmHg)	8.2 (6.6–10.2)	8.1 (6.5–9.9)	8.6 (6.9–10.7)	0.009
Vasoactive drugs, median				
Dopamine, *n* (%)	139 (11.1)	97 (10.8)	42 (11.9)	0.594
Dobutamine, *n* (%)	135 (10.8)	93 (10.4)	43 (12.1)	0.363
Norepinephrine, *n* (%)	979 (78.3)	689 (76.8)	290 (81.9)	0.048
Epinephrine, *n* (%)	91 (7.3)	68 (7.6)	23 (6.5)	0.506
Vasopressin, *n* (%)	31 (2.5)	24 (2.7)	7 (2.0)	0.474
VIS	35.5 (17.6–70.6)	35.7 (17.3–66.7)	33.9 (18.2–76.9)	0.445
Mechanical ventilation, *n* (%)	894 (71.5)	604 (67.3)	290 (81.9)	< 0.001
New CRRT, *n* (%)	312 (24.9)	165 (18.4)	147 (41.5)	< 0.001

*APACHE* II Acute Physiology and Chronic Health Evaluation II, *SOFA* Sequential Organ Failure Assessment, *COPD* Chronic Obstructive Pulmonary Disease, *mSBP*_*24h*_ mean Systolic Blood Pressure of the first 24 h after septic shock, *mDBP*_*24h*_ mean Diastolic Blood pressure of the first 24 h after septic shock, *mMAP*_*24h*_ mean Mean Artery Pressure of the first 24 h after septic shock, *mHR*_*24h*_ mean Heart Rate of the first 24 h after septic shock, *mCVP*_*24h*_ mean Centre Venous Pressure of the first 24 h after septic shock, *IQR* Interquartile Range, *VIS* Vasoactive-Inotropic Score, *CRRT* Continuous Renal Replacement Therapy

A total of 67 (5.4%) patients had a mDBP_24h_ < 50 mmHg, 386 (30.9%) patients had a mDBP_24h_ 50–60 mmHg, 512 (40.9%) patients mDBP_24h_ 60–70 mmHg, 238 (19.0%) patients mDBP_24h_ 70–80 mmHg, and 48 (3.8%) patients a mDBP_24h_ ≥ 80 mmHg. Compared with low mDBP_24h_ group patients, the high mDBP_24h_ group had less illness severity, decreased norepinephrine used proportion and VIS, and also had decreased levels of creatinine, fewer CRRT and mechanical ventilation (Table 2).

**Table 2:** Baseline characteristics of the study population according to different mDBP_24h_ levels

	Total *n* = 1251	mDBP_24h_ < 50 mmHg *n* = 67	mDBP_24h_ 50–60 mmHg *n* = 386	mDBP_24h_ 60–70 mmHg *n* = 512	mDBP_24h_ 70–80 mmHg *n* = 238	mDBP_24h_ ≥ 80 mmHg *n* = 48	*P*-value	χ^2^/*H*
Age, median (IQR)	68.0 (55.0–78.0)	76.0 (67.583.5)	74.0 (66.0–82.0)	65.0 (54.0–75.3)	62.0 (51.0–72.0)	50.5 (42.8–58.0)	0.255	5.326
Gender, Male, *n* (%)	845 (67.5)	41 (61.2)	246 (63.7)	340 (66.4)	182 (76.5)	36 (75.0)	0.007	13.964
APACHE II score, median (IQR)	19.0 (14.0–25.0)	23.0 (15.5–29.5)	20.0 (16.0–27.0)	18.0 (13.0–24.0)	19.5 (14.0–25.0)	15.5 (12.3–22.0)	0.006	14.298
SOFA score, median (IQR)	8.0 (5.0–10.0)	10.0 (6.0–12.8)	8.0 (6.0–10.0)	8.0 (5.0–10.0)	8.0 (5.0–10.0)	6.0 (4.0–9.0)	0.410	5.426
*Comorbidities, n (%)*								
Hypertension	657 (52.5)	43 (64.2)	220 (57.0)	247 (48.2)	116 (48.7)	31 (64.6)	0.005	14.674
Diabetes mellitus	336 (26.9)	23 (34.3)	108 (28.0)	141 (27.5)	52 (21.8)	12 (25.0)	0.249	5.396
Chronic congestive heart failure	237 (18.9)	17 (25.4)	88 (22.8)	87 (17.0)	39 (16.4)	1 (2.1)	0.002	16.852
Coronary heart disease	206 (16.5)	16 (23.9)	79 (20.5)	74 (14.5)	36 (15.1)	6 (12.5)	0.056	9.212
Chronic renal failure	93 (7.4)	7 (10.4)	38 (9.8)	30 (5.9)	10 (4.2)	8 (16.7)	0.004	15.548
COPD	86 (6.9)	5 (7.5)	31 (8.0)	27 (5.3)	22 (9.2)	1 (2.1)	0.153	6.701
Cirrhosis	25 (2.0)	3 (4.5)	12 (3.1)	7 (1.4)	2 (0.8)	1 (2.1)	0.125	7.206
Cancer (Solid tumor)	237 (18.9)	9 (13.4)	96 (24.9)	94 (18.4)	33 (13.9)	5 (10.4)	0.002	16.538
*Source of infection, n (%)*								
Lung	634 (50.7)	29 (43.3)	190 (49.2)	258 (50.4)	129 (54.2)	28 (58.3)	0.390	4.117
Abdomen	415 (33.2)	25 (37.3)	137 (35.5)	167 (32.6)	74 (31.1)	11 (22.9)	0.371	4.270
Blood	47 (3.8)	4 (6.0)	18 (4.7)	19 (3.7)	6 (2.5)	0	0.317	4.724
Skin	34 (2.7)	1 (1.5)	10 (2.6)	14 (2.7)	7 (2.9)	2 (4.2)	0.934	0.831
Urinary tract	89 (7.1)	5 (7.5)	23 (6.0)	40 (7.8)	17 (7.1)	5 (10.4)	0.749	1.931
Others	30 (2.4)	2 (3.0)	8 (2.1)	13 (2.5)	5 (2.1)	2 (4.2)	0.902	1.048
*Hemodynamic variables, median (IQR)*								
mSBP_24h_ (mmHg)	123.0 (116.6–131.1)	119.7 (112.9–124.5)	120.9 (114.7–128.0)	122.8 (116.1–130.3)	127.6 (121.6–134.2)	136.7 (129.2–145.8)	< 0.001	129.296
mDBP_24h_ (mmHg)	63.3 (57.6–69.3)	47.3 (43.6–48.8)	56.3 (54.0–58.4)	64.7 (62.5–67.1)	73.2 (71.5–75.7)	84.2 (81.6–87.4)	< 0.001	1127.930
mMAP_24h_ (mmHg)	83.1 (78.1–88.4)	70.1 (68.5–72.8)	77.7 (75.4–79.9)	84.1 (81.6–86.8)	91.6 (89.0–94.6)	102.1 (99.1–105.6)	< 0.001	884.859
mHR_24h_ (beats per minute)	94.6 (83.0–105.8)	91.9 (72.8–102.9)	91.8 (81.3–103.3)	95.3 (84.1–105.9)	98.0 (86.6–110.2)	96.3 (86.4–105.7)	< 0.001	24.695
mCVP_24h_ (mmHg)	8.2 (6.6–10.2)	7.4 (5.9–10.2)	8.2 (6.8–10.1)	8.2 (6.5–10.2)	8.5 (6.7–10.5)	8.7 (7.0–11.6)	0.551	3.042
Vasoactive drugs								
Dopamine, *n* (%)	139 (11.1)	11 (16.4)	45 (11.7)	53 (10.4)	27 (11.3)	3 (6.3)	0.480	3.488
Dobutamine, *n* (%)	135 (10.8)	8 (11.9)	45 (11.7)	9 (1.8)	23 (9.7)	1 (2.1)	< 0.001	42.034
Norepinephrine, *n* (%)	979 (78.3)	55 (82.1)	316 (81.9)	395 (77.1)	185 (77.7)	28 (58.3)	0.005	14.847
Epinephrine, *n* (%)	91 (7.3)	10 (14.9)	27 (7.0)	42 (8.2)	10 (4.2)	2 (4.2)	0.032	10.533
Vasopressin, *n* (%)	31 (2.5)	1 (1.5)	9 (2.3)	12 (2.3)	7 (2.9)	2 (4.2)	0.891	1.119
VIS, median (IQR)	35.5 (17.6–70.6)	37.4 (17.4–79.4)	35.7 (18.8–69.7)	35.7 (18.4–77.3)	33.3 (16.7–58.3)	25.0 (15.3–44.6)	0.054	9.303
Intravenous fluid administered^a^ (ml), median (IQR)	3937.2 (2968.5–5081.2)	4010.1 (3098.3–5195.7)	3944.1 (3014.0–5097.0)	3996.4 (3074.8–5127.0)	3684.6 (2730.0–4865.5)	3578.8 (2295.9–4656.9)	0.910	0.999
*Arterial blood gas analysis, median (IQR)*								
PH	7.3 (7.3–7.4)	7.4 (7.3–7.4)	7.3 (7.3–7.4)	7.3 (7.3–7.4)	7.4 (7.3–7.4)	7.4 (7.3–7.4)	0.038	10.135
Lactate (mmol/L)	2.5 (1.5–2.9)	2.5 (1.8–4.9)	2.5 (1.8–4.1)	2.6 (1.8–3.9)	2.4 (1.7–3.7)	2.1 (1.4–3.5)	0.232	5.590
Serum creatinine (mmol/L)	104.0 (72.0–185.8)	134.0 (83.5–226.0)	116.0 (80.5–195.0)	95.0 (68.0–169.0)	96.0 (62.3–158.5)	83.0 (62.0–132.0)	0.026	11.051
Length of stay in ICU (days), median (IQR)	8.3 (3.9–15.7)	6.7 (2.7–11.0)	7.4 (3.6–15.5)	8.0 (3.9–14.8)	10.7 (5.0–17.7)	9.6 (5.0–14.9)	0.157	6.633
Length of stay in Hospital (days), median (IQR)	17.6 (9.9–28.2)	12.8 (6.9–26.9)	16.9 (9.4–28.9)	18.4 (10.4–27.5)	18.2 (11.5–30.1)	18.4 (9.7–30.0)	0.013	12.585
Mechanical ventilation, *n* (%)	894 (71.5)	47 (70.1)	293 (75.9)	367 (71.7)	159 (66.6)	28 (58.3)	0.034	10.394
CRRT, *n* (%)	312 (24.9)	27 (40.3)	99 (25.6)	118 (23.0)	56 (23.5)	12 (25.0)	0.044	9.779

*APACHE* II Acute Physiology and Chronic Health Evaluation II, *SOFA* Sequential Organ Failure Assessment, *COPD* Chronic Obstructive Pulmonary Disease, *mSBP*_*24h*_ mean Systolic Blood Pressure of the first 24 h after septic shock, *mDBP*_*24h*_ mean Diastolic Blood pressure of the first 24 h after septic shock, *mMAP*_*24h*_ mean Mean Artery Pressure of the first 24 h after septic shock, *mHR*_*24h*_ mean Heart Rate of the first 24 h after septic shock, *mCVP*_*24h*_ mean Centre Venous Pressure of the first 24 h after septic shock, *IQR* Interquartile Range, *VIS* Vasoactive-Inotropic Score, *CRRT* Continuous Renal Replacement Therapy

^a^Intravenous fluid included crystalloids and colloid in the first 24 h after ICU admission of septic shock

### Association between mDBP_24h_ and 28-day mortality

**Fig. 2:**
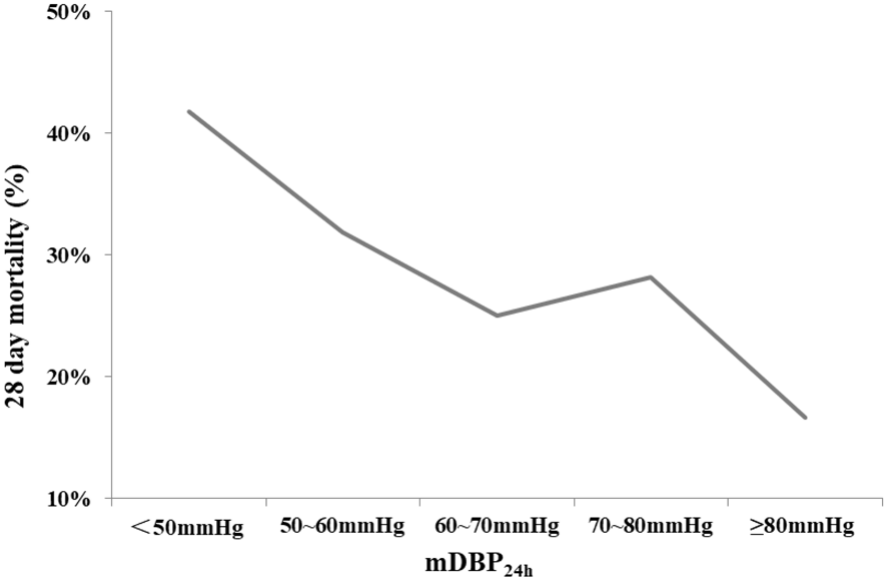
28-day mortality as a function of the mDBP_24h_ of septic shock controlled patients

**Fig. 3:**
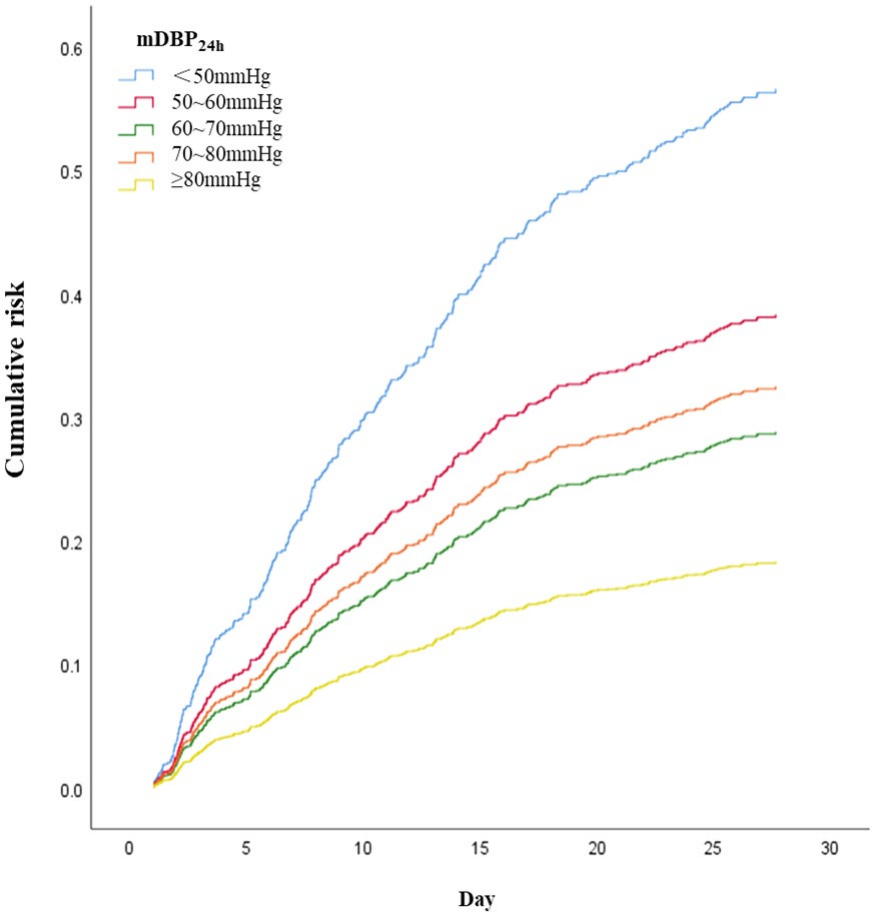
The mDBP_24h_ was inversely associated with 28 days mortality (unadjusted OR 0.814 per 10 mmHg higher mDBP_24h_, 95% CI 0.711–0.933, *P* = 0.003)

The 28-day mortality of the included septic shock patients was 28.3%. Crude 28-day mortality of septic shock patients was gradually decreased with the increased of mDBP_24h_ (41.8% vs. 31.9% vs. 25.0% vs. 28.2% vs. 16.7%, *P* = 0.006) (Fig. 2 and Additional file 2: Table S2). The mDBP_24h_ was inversely associated with 28-day mortality (unadjusted OR 0.814 per 10 mmHg higher mDBP_24h_, 95%CI 0.711–0.933, *P* = 0.003; optimal cutoff 58.9 mmHg) (Fig. 3). Similar findings were observed in the relationship of mDBP_24h_ with ICU mortality and hospital mortality (Additional file 2: Table S2, Additional file 6: Figure S1). The mDBP_24h_ was also inversely associated with ICU mortality (unadjusted OR 0.845 per 10 mmHg higher mDBP_24h_, 95%CI 0.735–0.971, *P* = 0.018; optimal cutoff 58.5 mmHg).

The patients were further divided into two groups according to whether the mDBP_24h_ was less than 59 mmHg. Compared with patients with mDBP_24h_ ≥ 59 mmHg, patients with mDBP_24h_ < 59 mmHg had older age, higher APACHE score, higher serum creatinine, lower levels of central venous oxygen saturation, more patients received mechanical ventilation and renal replacement therapy, and had higher ICU and hospital mortality (Additional file 3: Table S3). We analyzed the relationship between mDBP_24h_ and 28-day mortality in patients with mDBP_24h_ < 59 mmHg group. There is no correlation between DBP and mortality in the DBP impaired (mDBP_24h_ < 59 mmHg) group, OR 0.964 (95%CI 0.925–1.003, *P* = 0.073), which may be related to the small sample size.

**Fig. 4:**
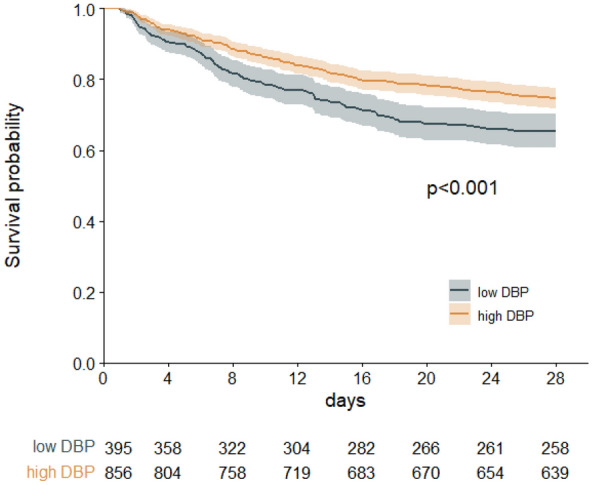
Probability of survival in septic shock patients through Day 28. The graph shows the Kaplan–Meier estimates for the probability of survival among septic shock patients with low DBP (mDBP_24h_ < 59 mmHg) and high DBP (mDBP_24h_ ≥ 59 mmHg) level. The *P*-value was calculated with the use of the log-rank test

The 28-day mortality of patients with mDBP_24h_ < 59 mmHg had an absolute risk reduction of 9.4% (*P* = 0.001) (Fig. 4). After multivariable adjustment, mDBP_24h_ < 59 mmHg remained inversely associated with 28-day mortality (adjusted OR 1.915, 95% CI 1.037–3.536, *P* = 0.038), while mMAP_24h_ and mSBP_24h_ was not associated with 28-day mortality (Table 3). The worst DBP in the first 24 h was also an independent factor of 28-day mortality (Additional file 4: Table S4).

**Table 3:** Predictors of 28-day mortality on multivariable regression

Variable	Adjusted OR	95%CI	*P*-value
Age (per year)	1.018	1.002–1.033	0.023
Gender	1.259	0.821–1.930	0.292
APACHE II	1.117	1.048–1.191	0.001
mDBP_24h_ < 59 mmHg	1.915	1.037–3.536	0.038
mMAP_24h_	1.005	0.958–1.053	0.849
mSBP_24h_	0.998	0.971–1.026	0.877
White blood cell	0.997	0.981–1.014	0.744
Platelet	1.000	0.998–1.002	0.995
Creatinine	1.000	1.000–1.000	0.779
Lactate	1.115	1.043–1.192	0.001
P/F ratio	0.996	0.994–0.999	0.003
VIS	1.001	0.998–1.004	0.636

*APACHE* II Acute Physiology and Chronic Health Evaluation II, *mDBP*_*24h*_ mean Diastolic Blood pressure of the first 24 h after septic shock, *mSBP*_*24h*_ mean Systolic Blood Pressure of the first 24 h after septic shock, *mMAP*_*24h*_ mean Mean Artery Pressure of the first 24 h after septic shock, *P/F ratio* Ratio of arterial partial oxygen pressure to inhaled oxygen concentration *VIS* Vasoactive-Inotropic Score

Compared with patients with mDBP_24h_ < 60 mmHg, the likelihood of 28-day survival rate of patients with mDBP_24h_ in 60–70 mmHg (OR 1.500 [1.134–1.984], *P* = 0.004) and great than or equal to 80 mmHg had all significantly increased (OR 2.500 [1.142–5.475], *P* = 0.022) (Table 4).

**Table 4:** Cumulative survival analysis of septic shock patients with different mDBP_24h_ levels

mDBP_24h_	*P*-value	OR (95% CI)
< 60 mmHg	Reference	–
60–70 mmHg	0.004	1.500 (1.134–1.984)
70–80 mmHg	0.164	1.276 (0.905–1.799)
≥ 80 mmHg	0.022	2.500 (1.142–5.475)

**Fig. 5:**
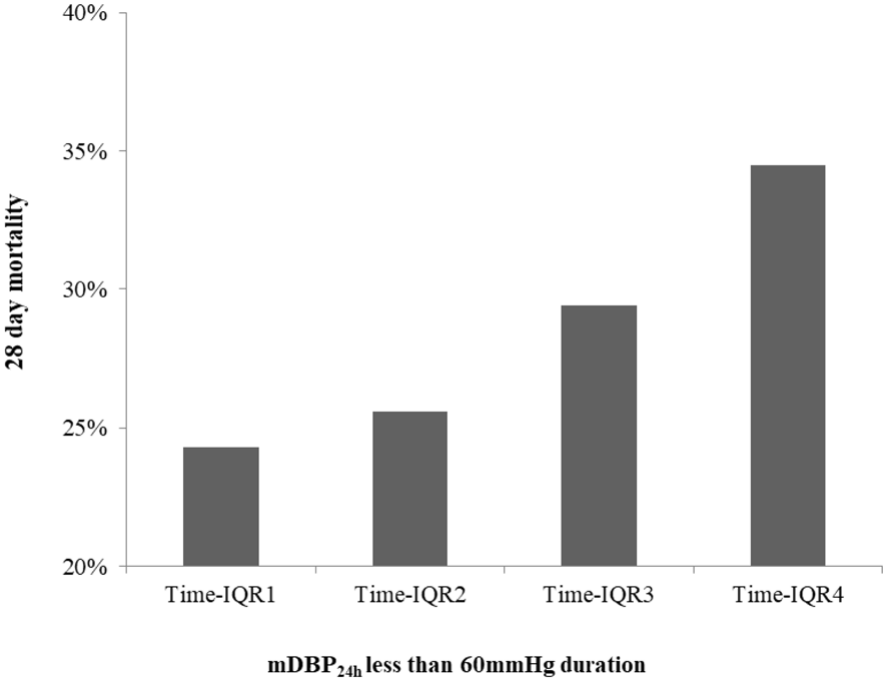
28-day mortality of different interquartile intervals with mDBP_24h_ less than 60 mmHg duration (Time-IQR1: ≤ 2 h, Time-IQR2: 2–8 h, Time-IQR3: 8–15 h, Time-IQR4: ≥ 15 h) IQR: interquartile range

The duration of mDBP_24h_ < 60 mmHg in the first 24 h was divided according to the interquartile range. Patients with septic shock in the first quartile had the lowest 28-day mortality, indicating that the longer duration of mDBP_24h_ < 60 mmHg, the higher the 28-day mortality was (*P* = 0.020) (Fig. 5). In contrast, the longer maintained mDBP_24h_ in 60–70 mmHg during the first 24 h, the lower the 28-day mortality was (*P* = 0.009) (Additional file 7: Figure S2). While in other mDBP_24h_ ranges, there was no significant difference in mortality among different mDBP_24h_ duration (Additional file 5: Table S5).

**Fig. 6:**
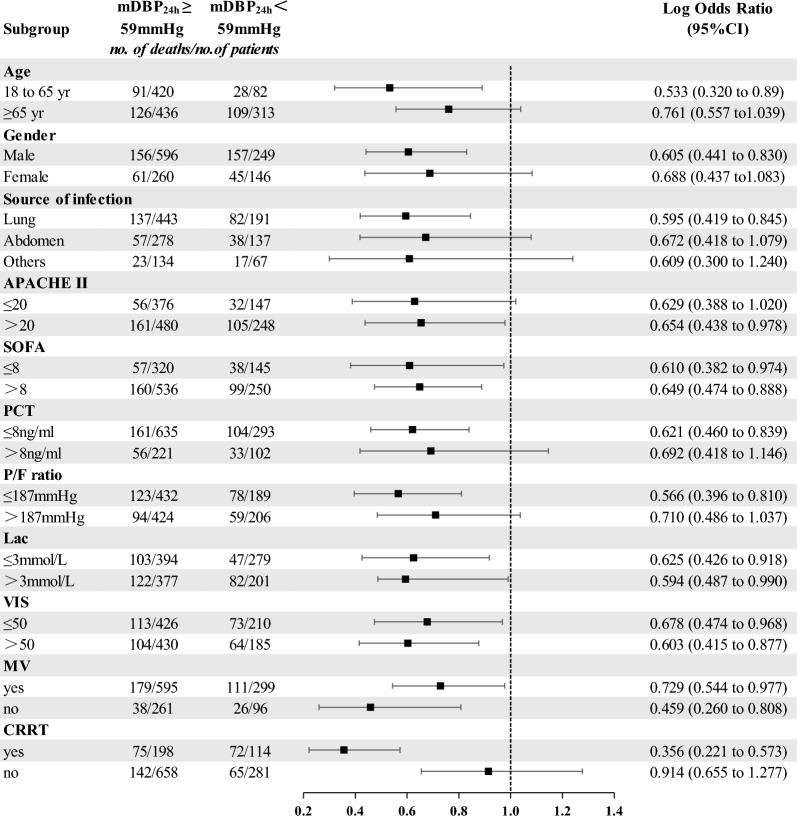
Subgroup analysis of the association between 28-day mortality and mDBP_24h_ ≥ 59 mmHg in septic shock patients

The subgroup analysis of relationship between mDBP_24h_ ≥ 59 mmHg and 28-day mortality showed that among the septic shocks patients who were younger than 65 years, underlying hypertension, APACHE II above 20, and P/F ratio less than or equal to 187 mmHg, mDBP_24h_ ≥ 59 mmHg was more relevant to 28-day survival (Fig. 6). Similar subgroup results were found in the relationship between mDBP_24h,_ mMAP_24h_ and 28-day survival, but not in mSBP_24h_ subgroup analysis (Additional file 8: Figure S3).

## Discussion

In this retrospective study of a large tertiary ICU septic shock controlled patients, we demonstrate that mDBP_24h_ is inversely associated with 28-day mortality. The septic shock controlled patients who were able to maintain a mDBP_24h_ great than or equal to 59 mmHg had lower 28-day mortality. Among those patients with a mDBP_24h_ blow 59 mmHg, they had more severely illness condition and higher 28-day mortality. These data also suggest that mDBP_24h_, not mMAP_24h_ and mSBP_24h_, was an independent predictor of 28-day mortality.

Surviving Sepsis Campaign guidelines recommended targeting a MAP of 65 mmHg in the initial resuscitation of septic shock patients [4]. However, even if septic shock patients get the MAP target above 65 mmHg, the 90-day mortality was still around 40% [8, 22]. Numerous studies have confirmed that there are still microcirculation disorders after shock resuscitation [23, 24]. DBP was the only independent microcirculatory determinant of tissue oxygen saturation resaturation (resStO_2_) [25], which was measured by Near infrared spectrometry as one of the main studied microcirculation parameters and strongly associated with outcome in sepsis patients [26, 27]. The findings of our study, which showed a higher DBP target (≥ 59 mmHg) in septic shock controlled patients was associated with lower lactate levels and better prognosis, may indirectly support the view of a correlation between DBP and microcirculation perfusion.

Vasodilation is an important pathophysiological feature and plays a key role in the progression of hypotension and tissue hypoperfusion in septic shock [28]. Considering DBP is a good marker of arterial tone, a low DBP in patients with septic shock detected at peripheral vessels should reflect the systemic vasodilation [10]. A retrospective cohort study indicated that DBP lower than 52 mmHg of nonsevere sepsis patients at emergence department(ED) triage (OR 4.59; 95% CI 1.57–13.39) was independently predict early progression to severe sepsis or septic shock within 96 h of ED presentation [11]. Other trials showed that low DBP was associated with the development of severe AKI [13]. And DBP within 24 h admission, not MAP, was a potential important hemodynamic target for preventing AKI in ICU patients [12]. Further studies proved that DBP, but not SBP, was one of the independent positive predictive factors of ICU patients’ outcome [14, 15, 29]. We also found that the serum creatinine was significantly higher in the low mDBP_24h_ group and a strong independent association between mDBP_24h_ and mortality.

Another physiological feature of DBP is a determinant of coronary perfusion. More than 50% of patients with septic shock have evidence of a reduced coronary flow blood reserve, which is a predictor of ICU mortality in septic shock [30]. A low DBP may impair the myocardial perfusion, especially in the case of tachycardia [31]. Our study showed that myoglobin was significantly higher in the low mDBP_24h_ group, suggesting that low DBP may be associated with myocardial ischemia. SPRINT data confirmed that a DBP lower than 50 mmHg was significantly associated with increased cardiovascular events in patients with 50 years older and a screening SBP of 130 to 180 mmHg [32]. And a DBP lower than 70 mmHg was significantly associated with mortality of patients with coronary artery disease [33]. A national cross-sectional survey showed that 39.6% and 17.0% of sepsis patients, respectively, had underlying hypertension and coronary artery disease in Chinese ICUs [3]. Therefore, a low DBP level during treatment for septic shock may significantly increase the risk of cardiovascular events.

The possible pathophysiological mechanisms of low DBP and high mortality are as follows: first, low DBP indicates more obvious vasodilation, which will lead to tissue hypoperfusion [10, 13]; second, low DBP will lead to reduced coronary blood flow and increase cardiovascular adverse events [30, 33]; the last one, low DBP is associated with impaired microcirculation [25]. Therefore, we need to further correct low DBP even after MAP target is achieved.

European Society of Intensive Care Medicine recommended that the combination of MAP (60–65 mmHg) and DBP (> 40 mmHg) targets should be considered as trigger to start vasopressor treatment in septic shock [17]. The flow diagram of initial resuscitation of sepsis induced hypotension or serum lactate ≥ 4 mmol/L suggested that we should initiated noradrenaline infusion along with the 30 ml/kg fluid bolus when DBP is lower than 50 mmHg [34]. However, these DBP cutoff values were either based of experts practice or the estimated value corresponded with a MAP of 65 mmHg and a SBP of 90 mmHg, and were not supported by clinical studies. Our findings supported that the mDBP_24h_ should be raised to a higher pressure level (≥ 59 mmHg) in the first 24 h of patients with septic shock controlled after initial resuscitation, showing it was associated with the illness severity and mortality benefit.

### Limitations

This retrospective study has a number of inherent limitations we should acknowledge. First, the missing data could bias the results and also other possible residual confounders due to single-center retrospective design, Second, patients were retrospectively enrolled from a single center which may impede the generalization of the results. The results of this single-center retrospective study need further studies to confirm. Third, this observational study could not lead to causal inferences for any associations. Finally, the mDBP_24h_ may include both noninvasive and invasive arterial pressure, and we could not distinguish the source of DBP measurements.

## Conclusions

There was an inverse correlation between DBP in the first 24 h and 28-day mortality among septic shock controlled patients admitted to the ICU. Patients with a mDBP_24h_ less than 59 mmHg during the first 24 h after septic shock controlled had an increased risk of 28-day mortality. These findings provide indirect support for a DBP target of 59 mmHg for septic shock controlled patient in ICU. More high quality prospective or randomized controlled studies are needed to further validate the DBP target in septic shock patients.

### Supplementary Information

**Additional file 1.** Percentages of missing data in the variables of interest in the cohort.

**Additional file 2.** Mortality of septic shock patients at different diastolic blood pressure levels.

**Additional file 3.** Baseline characteristics of the study population according to mDBP24h cutoff value.

**Additional file 4.** The association between the worst DBP and 28-day mortality.

**Additional file 5.** The relationship between the duration different mDBP24h levels and 28-day mortality of septicshock patients.

**Additional file 6.** Mortality of septic shock patients.

**Additional file 7.** The 28-day mortality of different interquartile intervals with mDBP24h in 60-70mmHg duration.

**Additional file 8.** Subgroup analysis of the association between 28 day survival and mDBP24h mSBP24h and mMAP24h in septic shock patients.

## Data Availability

The data in CDIC used and analyzed in the current study are available from the corresponding author on reasonable request.

## Abbreviations

AKI: Acute kidney injure
CDIC: Chinese Database in Intensive Care
DBP: Diastolic Blood Pressure
mDBP_24h_: Mean diastolic blood pressure during the first 24 h of septic shock diagnosis
MAP: Mean arterial pressure
VIS: Vasoactive-Inotropic Score
