# Supplementary material for: Association between diastolic blood pressure during the first 24 h and 28-day mortality in patients with septic shock: a retrospective observational study
Source: Eur J Med Res. 2023 Sep 9;28:329. doi: 10.1186/s40001-023-01315-z (PMC10492407; doi:10.1186/s40001-023-01315-z)
Supplement: Supplementary file 1 — Additional file 1. Percentages of missing data in the variables of interest in the cohort. [file 40001_2023_1315_MOESM1_ESM.docx]

Supplemental table 1 Percentages of missing data in the variables of interest in the cohort

| Variables | Cohort (n=1251) |
| --- | --- |
| Age | 0% |
| Male | 0% |
| APACHE II | 12.6% |
| SOFA | 13.9% |
| DBP | 1.6% |
| SBP | 1.5% |
| MAP | 1.1% |
| CVP | 4.4% |
| HR | 2.1% |
| PH | 0% |
| Lactate | 0% |
| Creatinine | 14.8% |
| PaO_2_/FiO_2_ ratio | 12.4% |
| White blood cell | 5.4% |
| Platelet | 3.5% |
| Hemoglobin | 5.1% |
| Alanine transaminase | 7.8% |
| Glutamic oxaloacetic transferase | 10.7% |
| Albumin | 19.1% |
| Troponin I | 12.8% |
| NT-pro BNP | 21.5% |
| Myoglobin | 15.1% |
| Activated prothrombin time | 14.4% |
| Prothrombin time | 20.1% |
| Fibrinogen | 18.5% |
| D-dimer | 14.8% |
| Procalcitonin | 12.8% |
| ScvO_2_ | 10.5% |
| Vasoactive drugs in the first 24h | 0% |

PACHE Ⅱ Acute Physiology and Chronic Health Evaluation Ⅱ, SOFA Sequential Organ Failure Assessment, SBP Systolic Blood Pressure, DBP Diastolic Blood pressure, MAP Mean Artery Pressure, HR Heart Rate, CVP Centre Venous Pressure, PaO_2_ partial pressure of oxygen, FiO_2_ fraction of inspired oxygen
