# Supplementary material for: Association between diastolic blood pressure during the first 24 h and 28-day mortality in patients with septic shock: a retrospective observational study
Source: Eur J Med Res. 2023 Sep 9;28:329. doi: 10.1186/s40001-023-01315-z (PMC10492407; doi:10.1186/s40001-023-01315-z)
Supplement: Supplementary file 2 — Additional file 2. Mortality of septic shock patients at different diastolic blood pressure levels. [file 40001_2023_1315_MOESM2_ESM.docx]

|  | Supplemental table2 Mortality of septic shock patients at different diastolic blood pressure levels | | | | | | | |
| --- | --- | --- | --- | --- | --- | --- | --- | --- |
| mDBP_24h_ | | Total n=1251 | ＜50mmHg  n=67 | 50~60mmHg  n=386 | 60~70mmHg  n=512 | 70~80mmHg  n=238 | ≥80mmHg  n=48 | p value |
| ICU mortality, n (%) | | 324 (25.9) | 24 (35.8) | 115 (29.8) | 114 (21.8) | 61 (25.6) | 10 (20.8) | 0.031 |
| Hospital mortality, n (%) | | 381 (30.5) | 29 (43.3) | 131 (33.9) | 137 (26.8) | 70 (29.4) | 14 (29.2) | 0.028 |
| 28d mortality, n (%) | | 354 (28.3) | 28 (41.8) | 123 (31.9) | 128 (25.0) | 67 (28.2) | 8 (16.7) | 0.006 |
