# Supplementary material for: Association between diastolic blood pressure during the first 24 h and 28-day mortality in patients with septic shock: a retrospective observational study
Source: Eur J Med Res. 2023 Sep 9;28:329. doi: 10.1186/s40001-023-01315-z (PMC10492407; doi:10.1186/s40001-023-01315-z)
Supplement: Supplementary file 3 — Additional file 3. Baseline characteristics of the study population according to mDBP24h cutoff value. [file 40001_2023_1315_MOESM3_ESM.docx]

| Supplemental table 3 Baseline characteristics of the study population according to mDBP_24h_ cutoff value | | | | |
| --- | --- | --- | --- | --- |
|  | Total n=1251 | mDBP_24h_≥59mmHg  n=856 | mDBP_24h_＜59mmHg  n=395 | p value |
| Gender, Male, n (%) | 845 (67.5) | 596 (69.6) | 249 (63.0) | 0.021 |
| Age, median(IQR) | 68.0 (55.0~78.0) | 65.0 (52.0~75.0) | 75.0 (67.0~82.0) | ＜0.001 |
| APACHE-Ⅱ | 19.0 (14.0~25.0) | 18.0 (13.0~24.0) | 21.0 (16.0~28.0) | ＜0.001 |
| **Vital signs** |  |  |  |  |
| mSBP_24h_ (mmHg) | 123.0 (116.6~131.1) | 124.4 (117.5~132.1) | 121.1 (114.9~128.0) | ＜0.001 |
| mDBP_24h_(mmHg) | 63.3 (57.6~69.3) | 66.8 (63.0~71.9) | 54.8 (51.4~57.3) | ＜0.001 |
| mMAP_24h_(mmHg) | 83.1 (78.1~88.4) | 86.1 (82.2~90.9) | 76.6 (73.6~79.2) | ＜0.001 |
| mCVP_24h_(mmHg) | 8.2 (6.6~10.2) | 8.3 (6.6~10.4) | 8.2 (6.6~10.0) | 0.460 |
| mHR_24h_(bpm) | 94.6 (83.0~105.8) | 96.2 (85.0~107.3) | 91.5 (80.1~103.0) | ＜0.001 |
| **Vasoactive agents use,** n (%) | | | | |
| Dopamineμ | 139 (11.1) | 88 (10.3) | 51 (12.9) | 0.169 |
| Dobutamin | 136 (10.9) | 90 (7.2) | 46 (11.6) | 0.550 |
| Epinephrine | 91 (7.2) | 61 (7.1) | 30 (7.6) | 0.767 |
| Noradrenaline | 979 (78.3) | 654 (76.4) | 325 (82.3) | 0.019 |
| Hypophysin | 31 (2.5) | 23 (2.7) | 8 (2.0) | 0.484 |
| **Vasoactive agents use (the maximum dose during the first 24 hours)** | | | | |
| Dopamine (μg·kg^-1^·min^-1^) | 10.0 (5.0~20.0) | 10.0 (5.0~20.0) | 10.0 (8.5~19.0) | 0.677 |
| Dobutamin (μg·kg^-1^·min^-1^) | 5.0 (3.0~5.0) | 5.0 (3.0~5.0) | 5.0 (4.0~5.8) | 0.277 |
| Epinephrine (μg·kg^-1^·min^-1^) | 0.04 (0.02~0.08) | 0.05 (0.03~0.10) | 0.03 (0.02~0.04) | 0.001 |
| Noradrenaline(μg·min^-1^) | 20.0 (10.0~40.0) | 20.0 (10.0~40.0) | 20.0 (10.0~45.0) | 0.879 |
| Hypophysin (U·h^-1^) | 2.0 (1.0~2.0) | 2.0 (1.0~2.0) | 2.0 (1.8~2.0) | 0.794 |
| VIS (μg·kg^-1^·min^-1^) | 35.3 (17.6~70.6) | 35.0 (17.3~71.2) | 35.7 (18.2~69.4) | 0.716 |
| **Blood test results during the first 24 hours** | | | | |
| White blood cell | 13.0 (8.9~18.7) | 13.0 (8.8~18.5) | 13.1 (9.1~19.2) | 0.424 |
| Platelet | 129.0 (74.0~186.0) | 132.0 (74.0~187.0) | 123.0 (71.0~184.0) | 0.581 |
| Hemoglobin | 106.0 (90.0~125.0) | 109.0 (92.0~128.0) | 102.0 (88.0~118.0) | ＜0.001 |
| Alanine transaminase | 40.0 (27.0~75.0) | 42.0 (27.0~82.0) | 37.0 (26.0~59.0) | 0.011 |
| Glutamic oxaloacetic transferase | 52.0 (30.0~111.5) | 54.5 (30.0~112.0) | 49.0 (29.0~109.0) | 0.246 |
| Albumin | 27.4 (22.7~30.5) | 27.8 (23.1~30.9) | 26.0 (20.9~29.4) | ＜0.001 |
| Blood urea nitrogen | 10.8 (6.9~16.8) | 10.2 (6.5~16.1) | 11.6 (7.5~19.6) | 0.002 |
| Serum creatinine | 105.0 (72.0~185.8) | 99.0 (69.0~174.0) | 119.0 (81.0~204.0) | 0.001 |
| Troponin I | 0.05 (0.02~0.20) | 0.05 (0.02~0.19) | 0.04 (0.02~0.22) | 0.596 |
| NT-pro BNP | 2240.0 (759.0~6370.0) | 1935.0 (650.3~5552.5) | 3240.0 (1090.0~8400.0) | ＜0.001 |
| Myoglobin | 241.0 (107.0~498.0) | 228.5 (100.3~479.5) | 270.0 (139.0~560.3) | 0.004 |
| Activated prothrombin time | 35.4 (31.2~42.0) | 35.2 (30.9~41.8) | 36.1 (31.6~43.8) | 0.038 |
| Prothrombin time | 14.6 (13.2~17.2) | 14.5 (13.1~16.9) | 14.7 (13.4~17.6) | 0.043 |
| Fibrinogen | 3.9 (3.2~4.6) | 4.0 (3.3~4.7) | 3.8 (3.1~4.4) | ＜0.001 |
| D-dimer | 1751.0 (679.0~3579.5) | 1691.0 (641.8~3561.5) | 1905.5 (736.5~3610.0) | 0.425 |
| Procalcitonin | 3.6 (1.0~13.0) | 3.5 (1.0~13.1) | 4.0 (1.0~12.7) | 0.824 |
| C-reactive protein | 126.0 (64.8~191.0) | 131.5 (68.1~193.5) | 117.5 (60.7~175.5) | 0.049 |
| P/F ratio, (mmHg) | 168.5 (113.1~240.7) | 168.5 (112.2~238.0) | 169.2 (118.5~248.3) | 0.508 |
| Lactate, (mmol/L) | 2.5 (1.8~3.9) | 2.5 (1.8~3.8) | 2.5 (1.8~4.1) | 0.218 |
| ScvO_2_, (%) | 76.7 (70.9~81.6) | 77.0 (71.5~82.5) | 75.3 (69.3~80.4) | 0.001 |
| **Secondary Prognosis** |  |  |  |  |
| Mechanical ventilation, n(%) | 894 (71.5) | 595 (69.5) | 299 (75.7) | 0.024 |
| CRRT, n(%) | 312 (24.9) | 198 (23.1) | 114 (28.9) | 0.029 |
| Hospital duration (day) | 17.6 (9.9~28.2) | 18.1 (10.7~28.0) | 16.6 (8.9~28.6) | 0.072 |
| ICU duration (day) | 8.3 (3.9~15.7) | 8.9 (4.4~16.0) | 7.1 (3.5~14.6) | 0.007 |
| ICU mortality, n(%) | 324 (25.9) | 197(20.0) | 127(32.2) | 0.001 |
| Hospital mortality, n(%) | 381 (30.5) | 236(27.8) | 146(37.0) | 0.001 |

*APACHE Ⅱ* Acute Physiology and Chronic Health Evaluation Ⅱ, *mSBP_24h_* mean Systolic Blood Pressure of the first 24h after septic shock, *mDBP_24h_* mean Diastolic Blood pressure of the first 24h after septic shock, *mMAP_24h_* mean Mean Artery Pressure of the first 24h after septic shock, *mHR_24h_* mean Heart Rate of the first 24h after septic shock, *mCVP_24h_* mean Centre Venous Pressure of the first 24h after septic shock, IQR Interquartile Range, *VIS* Vasoactive-Inotropic Score, *P/F ratio* Ratio of Partial Oxygen Pressure to Fraction of Inspired Oxygen, *CRRT* Continuous Renal Replacement Therapy, *ICU* Intensive Care Unit.

Definition of abbreviations: SBP=Systolic Blood Pressure; DBP=Diastolic Blood pressure; MAP=Mean Artery Pressure; HR=Heart Rate; CVP=Centre Venous Pressure; APACHE= Acute Physiology and Chronic Health Evaluation; IQR= interquartile range; SOFA= sequential organ failure assessment; VIS= Vasoactive-Inotropic Score; P/F ratio= ratio of PaO2 to FiO2; ScvO2=Central venous oxygen saturation; CRRT= continuous renal replacement therapy.
