# Supplementary material for: Association between diastolic blood pressure during the first 24 h and 28-day mortality in patients with septic shock: a retrospective observational study
Source: Eur J Med Res. 2023 Sep 9;28:329. doi: 10.1186/s40001-023-01315-z (PMC10492407; doi:10.1186/s40001-023-01315-z)
Supplement: Supplementary file 4 — Additional file 4. The association between the worst DBP and 28-day mortality. [file 40001_2023_1315_MOESM4_ESM.docx]

| Supplemental table 4 the association between the worst DBP and 28 day mortality | | | | | | | |
| --- | --- | --- | --- | --- | --- | --- | --- |
| Univariate analysis | | | | Multivariate analysis | | | |
| variable | OR | 95%CI | P value | Variable | OR | 95%CI | P value |
| DBPmin | 0.971 | 0.958-0.984 | 0.000 | DBPmin | 0.975 | 0.955-0.997 | 0.023 |
|  |  |  |  | Age | 1.008 | 0.999-1.018 | 0.094 |
|  |  |  |  | Gender | 0.840 | 0.632-1.115 | 0.228 |
|  |  |  |  | APACHEⅡ | 1.092 | 1.070-1.114 | 0.000 |
|  |  |  |  | mMAP24h | 1.014 | 0.981-1.049 | 0.404 |
|  |  |  |  | mSBP24h | 0.990 | 0.970-1.009 | 0.300 |
|  |  |  |  | White blood cell | 0.993 | 0.980-1.007 | 0.323 |
|  |  |  |  | Procalcitonin | 0.989 | 0.974-1.005 | 0.193 |
|  |  |  |  | VIS | 1.092 | 1.070-1.114 | 0.000 |

*APACHE Ⅱ* Acute Physiology and Chronic Health Evaluation Ⅱ, *DBPmin* minimum diastolic blood pressure of the first 24h after septic shock, *mDBP_24h_* mean Diastolic Blood pressure of the first 24h after septic shock, *mSBP_24h_* mean Systolic Blood Pressure of the first 24h after septic shock, *mMAP_24h_* mean Mean Artery Pressure of the first 24h after septic shock, *VIS* Vasoactive-Inotropic Score
