# Supplementary material for: Association between diastolic blood pressure during the first 24 h and 28-day mortality in patients with septic shock: a retrospective observational study
Source: Eur J Med Res. 2023 Sep 9;28:329. doi: 10.1186/s40001-023-01315-z (PMC10492407; doi:10.1186/s40001-023-01315-z)
Supplement: Supplementary file 5 — Additional file 5. The relationship between the duration different mDBP24h levels and 28-day mortality of septicshock patients. [file 40001_2023_1315_MOESM5_ESM.docx]

| mDBP_24h_ | 28 day mortality n (%) | | | | p value |
| --- | --- | --- | --- | --- | --- |
|  | Time-IQR1 | Time-IQR2 | Time-IQR3 | Time-IQR4 |  |
| ＜60mmHg | 78/321(24.3) | 88/344(25.6) | 82/279(29.4) | 106/307(34.5) | 0.020 |
| 60~70mmHg | 118/351(33.6) | 99/337(29.4) | 76/280(27.1) | 61/283(21.6) | 0.009 |
| 70~80mmHg | 134/435(30.8) | 66/221(29.9) | 80/292(27.4) | 74/303(24.4) | 0.264 |
| ≥80mmHg | 171/602(28.4) | 56/216(25.9) | 45/169(26.6) | 82/264(31.1) | 0.609 |

Supplemental table 5 The relationship between the duration different mDBP_24h_ levels and 28 day mortality of septic shock patients.
