# Supplementary figures and images for: Association between diastolic blood pressure during the first 24 h and 28-day mortality in patients with septic shock: a retrospective observational study
Source: Eur J Med Res. 2023 Sep 9;28:329. doi: 10.1186/s40001-023-01315-z (PMC10492407; doi:10.1186/s40001-023-01315-z)

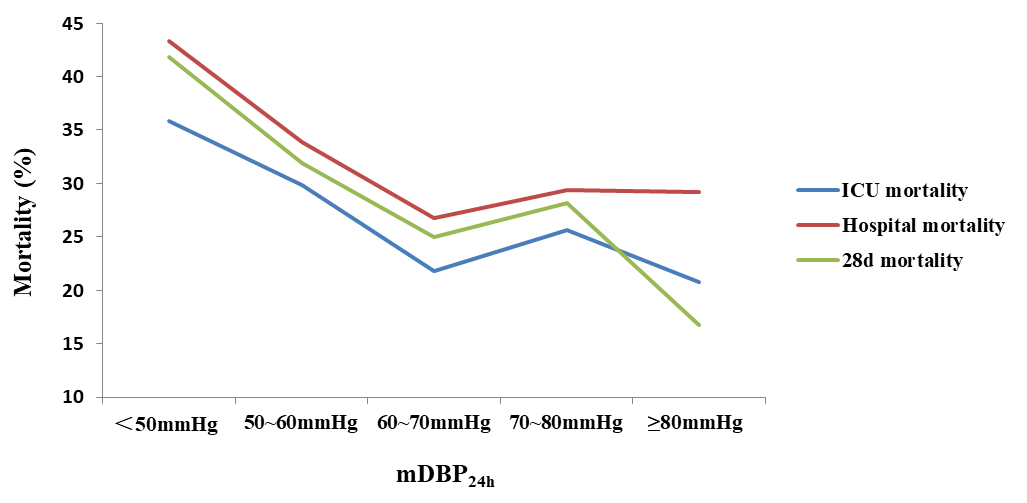


Supplemental figure 1 mortality of septic shock patients

Supplement: Supplementary file 6 — Additional file 6. Mortality of septic shock patients. [file 40001_2023_1315_MOESM6_ESM.docx]
