# Supplementary material for: Association between diastolic blood pressure during the first 24 h and 28-day mortality in patients with septic shock: a retrospective observational study
Source: Eur J Med Res. 2023 Sep 9;28:329. doi: 10.1186/s40001-023-01315-z (PMC10492407; doi:10.1186/s40001-023-01315-z)
Supplement: Supplementary file 7 — Additional file 7. The 28-day mortality of different interquartile intervals with mDBP24h in 60-70mmHg duration. [file 40001_2023_1315_MOESM7_ESM.docx]

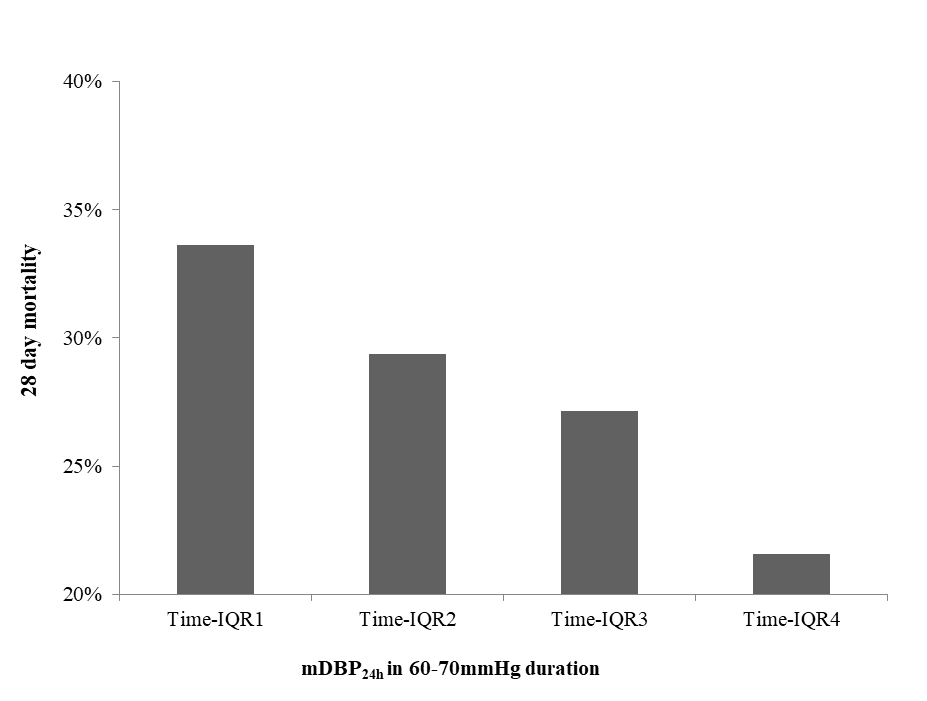


Supplemental Figure 2. 28 day mortality of different interquartile intervals with mDBP_24h_ in 60-70mmHg duration (Time-IQR1: ≤4 hours, Time-IQR2: 4-8hours, Time-IQR3: 8-11hours, Time-IQR4: ≥11hours ); IQR: interquartile range.
