# Supplementary material for: Association between diastolic blood pressure during the first 24 h and 28-day mortality in patients with septic shock: a retrospective observational study
Source: Eur J Med Res. 2023 Sep 9;28:329. doi: 10.1186/s40001-023-01315-z (PMC10492407; doi:10.1186/s40001-023-01315-z)
Supplement: Supplementary file 8 — Additional file 8. Subgroup analysis of the association between 28 day survival and mDBP24h mSBP24h and mMAP24h in septic shock patients. [file 40001_2023_1315_MOESM8_ESM.docx]

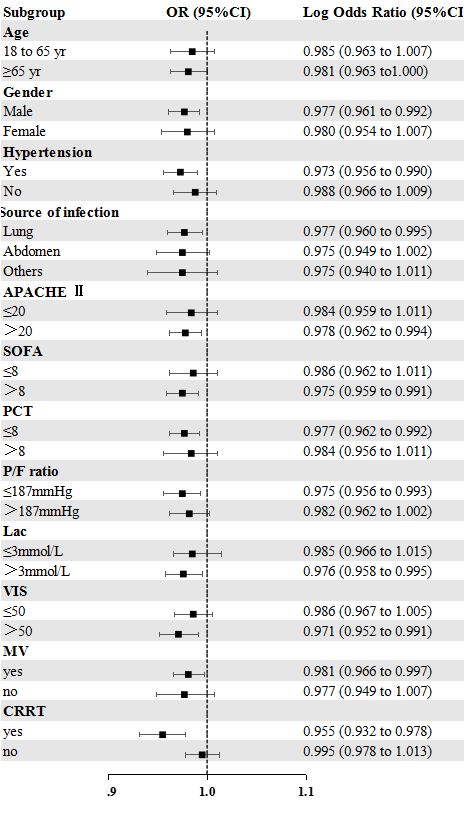


Supplemental figure 3a Subgroup analysis of the association between 28 day survival and mDBP_24h_ in septic shock patients


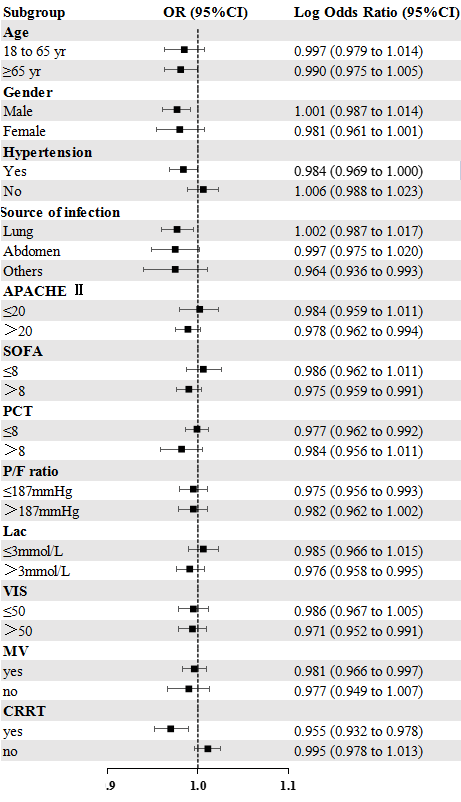


Supplemental figure 3b Subgroup analysis of the association between 28 day survival and mSBP_24h_ in septic shock patients


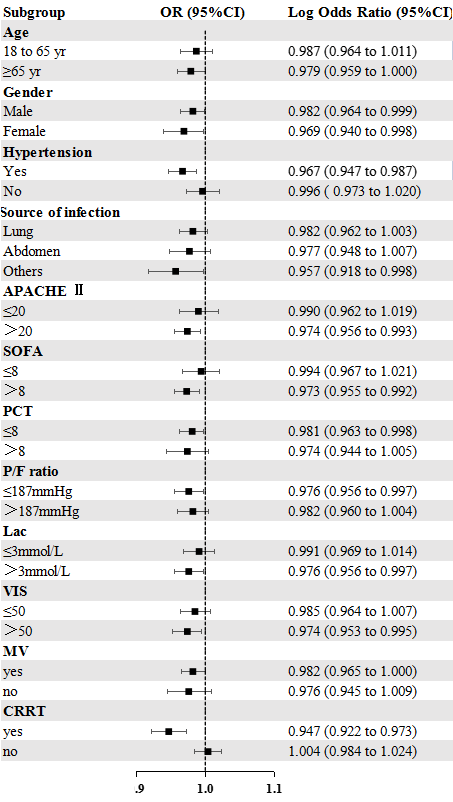


Supplemental figure 3c Subgroup analysis of the association between 28 day survival and mMAP_24h_ in septic shock patients
